# P-1982. SARS-CoV-2 Rebound during Acute Infection is Associated with Long COVID

**DOI:** 10.1093/ofid/ofae631.2140

**Published:** 2025-01-29

**Authors:** Nisha Ramdeep, Carly Herbert, Annukka A R Antar, Apurv Soni, Yukari C Manabe

**Affiliations:** Johns Hopkins University School of Medicine, Baltimore, Maryland; UMass Chan Medical School, Worcester, Massachusetts; Johns Hopkins University School of Medicine, Baltimore, Maryland; UMass Chan Medical School, Worcester, Massachusetts; Johns Hopkins University School of Medicine, Baltimore, Maryland

## Abstract

**Background:**

SARS-CoV-2 rebound, defined as recurrence of a positive SARS-CoV-2 test after testing negative during an acute infection, has received attention in patients treated with nirmatrelvir/ritonavir. We examined the association between SARS-CoV-2 rebound in untreated patients with delayed viral clearance and subsequent self-reported long COVID.

**Figure:**
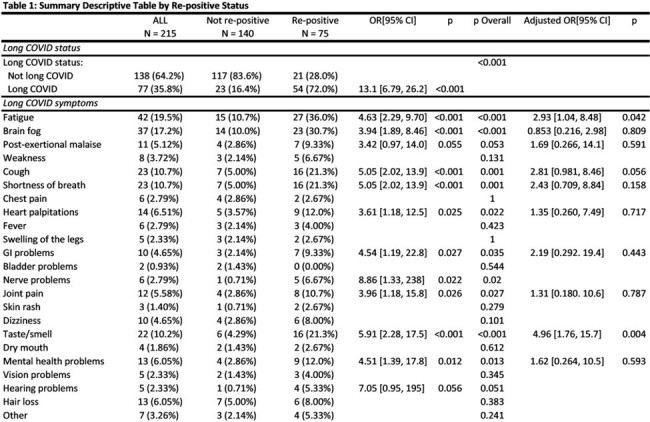

**Methods:**

From October 2021 to February 2022, the Test Us at Home (TUAH) study enrolled 7361 participants to self-collect anterior nasal specimens for SARS-CoV-2 RT-PCR testing every 24-48 hours for 10-14 days. In August 2023, 244 participants with ≥ 1 positive test during the original study period were surveyed about long COVID symptoms, defined as new symptoms persisting for 3+ months post-infection. Viral rebound was defined as testing positive, negative, and subsequently positive again during the 10-14 day study period. Only participants with ≥ 3 molecular (RT-PCR) tests were included (Figure 1). The associations between rebound (outcome) and presence of long COVID (exposure 1) as well as different symptoms of long COVID (exposure 2) were examined using separate multivariable logistic regression models that adjusted for participant-level confounding variables.

**Figure:**
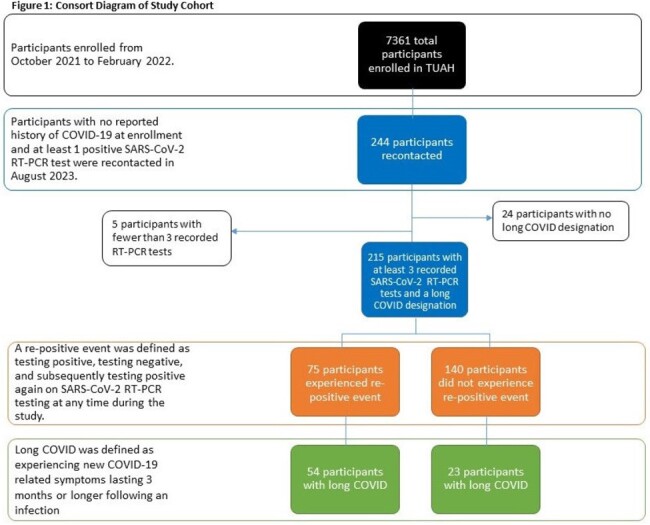

**Results:**

Data from 215 eligible participants were analyzed; 77 (35.8%) had long COVID. 75 of 215 (34.9%) experienced rebound. Long COVID was associated with 13-fold higher odds of experiencing viral rebound during acute infection (OR: 13.1, 95% CI: [6.79, 26.2]). When adjusting for other symptoms and covariates, there was an association between viral rebound and 1) post-acute fatigue (OR: 2.93, 95% CI: [1.04, 8.48]) and 2) taste or smell changes (OR: 4.96, 95% CI [1.76, 15.7]). There were no notable associations in demographics or pre-existing conditions with viral rebound.

**Conclusion:**

Acutely infected COVID-19 participants who experienced viral RNA rebound in the first 14 days after symptom onset were significantly more likely to have long COVID with fatigue or changes in taste or smell.

**Disclosures:**

Yukari C. Manabe, MD, Cepheid: Research materials to JHU|Chembio: Research materials to JHU|Hologic: Research grant to JHU|Roche: Research materials to JHU

